# Sociodemographic inequalities in mortality from drowning in the Baltic countries and Finland in 2000–2015: a register-based study

**DOI:** 10.1186/s12889-023-15999-9

**Published:** 2023-06-07

**Authors:** Andrew Stickley, Aleksei Baburin, Domantas Jasilionis, Juris Krumins, Pekka Martikainen, Naoki Kondo, Jae Il Shin, Yosuke Inoue, Mall Leinsalu

**Affiliations:** 1 Stockholm Centre for Health and Social Change, Södertörn University, Huddinge, 141 89 Sweden; 2 Department of Social Epidemiology, Graduate School of Medicine, School of Public Health, Kyoto University, Kyoto, Japan; 3 Department of Epidemiology and Biostatistics, National Institute for Health Development, Tallinn, Estonia; 4 Max Planck Institute for Demographic Research, Rostock, Germany; 5 Demographic Research Centre, Vytautas Magnus University, Kaunas, Lithuania; 6 Demography Unit, Faculty of Business, Management and Economics, University of Latvia, Riga, Latvia; 7 Population Research Unit, University of Helsinki, Helsinki, Finland; 8 Department of Pediatrics, Yonsei University College of Medicine, Yonsei-ro 50, Seodaemun- gu, Seoul, Korea; 9 Department of Epidemiology and Prevention, Center for Clinical Sciences, National Center for Global Health and Medicine, Tokyo, Japan

**Keywords:** Alcohol, Death, Drowning, Education, Inequality, Urban-rural

## Abstract

**Background:**

Drowning is an important public health problem. Some evidence suggests that the risk of drowning is not distributed evenly across the general population. However, there has been comparatively little research on inequalities in drowning mortality. To address this deficit, this study examined trends and sociodemographic inequalities in mortality from unintentional drowning in the Baltic countries and Finland in 2000–2015.

**Methods:**

Data for Estonia, Latvia and Lithuania came from longitudinal mortality follow-up studies of population censuses in 2000/2001 and 2011, while corresponding data for Finland were obtained from the longitudinal register-based population data file of Statistics Finland. Deaths from drowning (ICD-10 codes W65–W74) were obtained from national mortality registries. Information was also obtained on socioeconomic status (educational level) and urban-rural residence. Age-standardised mortality rates (ASMRs) per 100 000 person years and mortality rate ratios were calculated for adults aged 30–74 years old. Poisson regression analysis was performed to assess the independent effects of sex, urban-rural residence and education on drowning mortality.

**Results:**

Drowning ASMRs were significantly higher in the Baltic countries than in Finland but declined by nearly 30% in all countries across the study period. There were large inequalities by sex, urban-rural residence and educational level in all countries during 2000–2015. Men, rural residents and low educated individuals had substantially higher drowning ASMRs compared to their counterparts. Absolute and relative inequalities were significantly larger in the Baltic countries than in Finland. Absolute inequalities in drowning mortality declined in all countries across the study period except between urban and rural residents in Finland. Changes in relative inequalities were more variable during 2000–2015.

**Conclusion:**

Despite a sharp reduction in deaths from drowning in the Baltic countries and Finland in 2000–2015, drowning mortality was still high in these countries at the end of the study period with a substantially larger risk of death seen among men, rural residents and low educated individuals. A concerted effort to prevent drowning mortality among those most at risk may reduce drownings considerably in the general population.

**Supplementary Information:**

The online version contains supplementary material available at 10.1186/s12889-023-15999-9.

## Introduction

Drowning, which has been defined as “the process of experiencing respiratory impairment from submersion/immersion in liquid” is a serious public health threat [1]. In 2019 it was estimated that 236 000 people died worldwide as a result of drowning, with these deaths comprising 7% of all injury-related deaths [2]. There has been a sharp reduction in drowning deaths in recent years, with data from the Global Burden of Disease 2017 Study showing that unintentional drowning mortality fell by over 40% globally between 1990 and 2017 but with inconsistencies observed across countries [3]. However, it has been suggested that global mortality statistics may vastly underestimate the public health burden related to drowning due to the way drowning deaths are categorised [2]. In addition to its public health impact, the economic burden of drowning is also considerable. It has been estimated that the annual cost of coastal drownings is in excess of $273 million in the United States [4], while a recent study from Australia estimated that the average yearly cost of unintentional fatal drownings was 1.24 billion Australian dollars in the period from 2002 to 2017 [5].

Research suggests that the risk of drowning is not distributed evenly across the general population. In particular, a large body of evidence has emerged that males are more likely to drown than females [2, 6–8], which may be related to a range of factors including a greater propensity to engage in risk-taking behaviours such as swimming in natural bodies of water and consuming alcohol while swimming [9]. Similarly, there are also geographical differences in drowning. Studies from high-income countries [10, 11] have indicated that rurality is associated with higher mortality from unintentional drowning, which in Europe, has been linked to the proximity of natural bodies of water, poorer access to emergency services, and poverty [12]. The latter ties in with other research which has highlighted that there are socioeconomic differences in drowning mortality. For example, in Europe, research has indicated an increased mortality risk from unintentional drowning among deprived people [12], while a study from Denmark found that having a lower social position (i.e. as indicated by being outside the labour market) was also linked to a higher mortality rate from drowning [13].

Against this backdrop the aim of the current study is to examine trends and sociodemographic inequalities in mortality from unintentional drowning in the Baltic countries (Estonia, Latvia, Lithuania) and Finland in 2000–2015. There are several factors motivating this research. First, although there is some evidence that unintentional drowning deaths may have fallen sharply in the Baltic countries in recent years [14], even at the end of our study period (2016) these countries continued to have comparatively high drowning mortality rates – occupying three of the top four positions in the European Union (EU) in terms of the standardised number of deaths per 100 000 residents, while Finland also ranked well above the EU average [15]. Second, although research has been previously undertaken on drowning in Finland [16–18], as yet, there has been a lack of systematic research on this topic in the Baltic countries. This is an important omission given these countries’ high drowning mortality rates and the recent call to better quantify the drowning burden in Europe including its context [19]. In particular, little is known about urban-rural differences in drowning mortality in these countries even though a recent study found that there were significant differences in urban-rural all-cause mortality across these four countries [20]. Third, to the best of our knowledge there has been an absence of research on socioeconomic inequalities in drowning mortality in the Baltic countries and little research on this phenomenon in other European countries more generally. As earlier research linked low education to an elevated drowning mortality risk in Canada [21], while recent studies have reported educational inequalities in several causes of death in the Baltic countries and Finland [22–26], it is possible that socioeconomic differences might also play a role in drowning mortality in this setting. Finally, little evidence on changes in inequalities in drowning mortality exists. Thus, it is not clear whether the recent declines in overall drowning mortality observed in many high-income countries are accompanied by increasing or declining inequalities.

Thus, this study has several components. First, we will assess trends in overall drowning mortality in the Baltic countries and Finland in the period 2000–2015. Then we will evaluate trends in drowning mortality by sex, urban-rural residence and educational level and how these changes affected both absolute and relative inequalities in drowning mortality across the study period.

## Methods

### Data

Data for Estonia, Latvia and Lithuania were obtained from longitudinal mortality follow-up studies of population censuses in 2000 (2001 in Lithuania) and 2011 involving all permanent residents. The censuses in the Baltic countries combined traditional survey-based enumeration (the share of coverage ranged from 91% in Latvia to 98% in Estonia) and register-based enumeration [27]. The register-based data did not include information about socioeconomic status and were therefore excluded from the analysis. All individuals were followed from the census date until the date of death or emigration, or until the end of the follow-up period. The date and cause of death were linked from national mortality registries. All data linkages were performed by national statistical offices. Corresponding data for Finland were obtained from the longitudinal register-based population data file of Statistics Finland covering the total population. For this study data were organised into two sub-periods: 2000–2007 and 2008–2015. To ensure that a complete educational history was obtained and to achieve optimal accuracy of cause of death ascertainment, this study focuses on those aged 30–74 years. Population exposures were estimated by adding up the number of person years lived by individuals within each 5-year interval age group in a given period. Deaths were allocated to age intervals according to age at death. Data were anonymised and aggregated into multidimensional frequency tables combining deaths and population exposures split by study period and sociodemographic variables before they were released for research purposes.

### Measures

Deaths from drowning were classified as W65–W74 (accidental drowning and submersion) using the 10th revision of the International Classification of Diseases (ICD-10). Sociodemographic data are census-based and were harmonised following the common study protocol. Socioeconomic status was assessed using data on educational level, which was categorised using the International Standard Classification of Education (ISCED) 2011 [28]. *Low* education refers to primary and lower secondary education (ISCED categories 0–2), *middle* education includes upper secondary and post-secondary non-tertiary education (categories 3–4), and *high* education covers tertiary education (categories 5–8). Urban-rural residence was defined using national administrative classifications. In the Baltic countries, settlements with more than 2000 inhabitants (3000 in Lithuania) are considered urban. In Finland, an urban settlement is defined as a cluster of dwellings with at least 200 inhabitants per 250 × 250 square metres. Study subjects with missing values comprised less than 1% of all subjects and these cases were excluded from the analysis.

### Statistical analysis

To assess the trends and inequalities in mortality from drowning, age-standardised mortality rates (ASMRs) per 100 000 person years and mortality rate ratios (RR) together with 95% confidence intervals (CI) were calculated using the European Standard Population [29]. Additionally, multivariate Poisson regression was applied to assess the independent effect of sex, urban-rural residence and education on mortality from drowning. Adjusted mortality rate ratios were calculated for both periods. Statistical analyses were performed using STATA 14.2 (Stata Corp., College Station, Texas, USA).

## Results

This study included 7098 deaths from drowning and approximately 104 million person years (Table 1). The percentage of urban residents was higher in Finland compared to the Baltic countries. In all countries, the proportion of urban and rural residents remained approximately the same over the study period. In 2000–2015, the share of higher educated individuals increased in all countries but was substantially lower in rural areas (data not shown). In 2000–2007, drowning ASMRs per 100 000 person years were much higher in the Baltic countries (ranging from 7.7 in Estonia to 12.7 in Lithuania) than in Finland (3.6). Between 2000–2007 and 2008–2015, ASMRs declined by nearly 30% in all countries.

**Table 1:** Characteristics of the study populations, age-standardised mortality rate per 100 000 person years and mortality rate ratios for drownings in 2000–2015 in the 30–74 age group, total population

Country	Period	Deaths N	Person years N	Educational level			Urban population %	ASMR (95% CI)	RR (95% CI)
				High %	Middle %	Low %			
Finland	2000–2007	864	23,431,363	29.1	37.7	33.2	83.0	3.6 (3.3–3.8)	1
	2008–2015	673	24,074,431	32.6	42.3	25.1	84.3	2.5 (2.3–2.7)	0.70 (0.63–0.78)
Estonia	2000–2007	439	5,679,250	30.7	46.9	22.4	64.9	7.7 (7.0–8.5)	1
	2008–2015	317	5,767,086	34.5	48.7	16.8	64.7	5.4 (4.8–6.0)	0.70 (0.60–0.81)
Latvia	2000–2007	1100	9,062,656	17.5	58.7	23.8	68.4	12.3 (11.6–13.1)	1
	2008–2015	775	8,781,861	22.6	61.2	16.1	68.3	8.8 (8.2–9.4)	0.71 (0.65–0.78)
Lithuania	2001–2007	1617	12,854,909	17.9	59.8	22.3	68.4	12.7 (12.0–13.3)	1
	2008–2015	1313	14,140,553	22.3	60.9	16.9	69.0	9.2 (8.7–9.8)	0.73 (0.68–0.79)

The follow up in the 1st period started from the census date in the Baltic countries, i.e. 31.03.2000 in Estonia, 1.03.2000 in Latvia, and 6.04.2001 in Lithuania; in 2008 the follow up started on January 1; the follow up ended on December 31 in respective periods

ASMR, age-standardised mortality rate per 100 000 person years; CI, confidence interval; RR, mortality rate ratio between two periods

Trends and inequalities in drowning mortality by sex, urban-rural residence and educational level are presented in Table 2. In 2000–2007, ASMRs were considerably larger among men than women; mortality rate ratios ranged from 5.3 in Finland to 6.6 in Estonia. Between 2000–2007 and 2008–2015, in Finland and Estonia the mortality decline was larger among men in both absolute and relative terms resulting in smaller absolute and relative inequalities. In Latvia and Lithuania, the relative decline was larger among women resulting in increasing rate ratios, although absolute inequalities also declined.

**Table 2:** Age-standardised mortality rate per 100 000 person years and mortality rate ratios for drownings in 2000–2015 in the 30–74 age group

		ASMR (95% CI)			RR (95% CI)	
		2000–2007	2008–2015	Change (%)	2000–2007	2008–2015
Finland	Women	1.1 (0.9–1.3)	0.9 (0.8–1.1)	-0.2 (-19.3)	1	1
	Men	6.1 (5.6–6.5)	4.1 (3.8–4.5)	-1.9 (-32.0)	5.32 (4.44–6.42)	4.51 (3.70–5.52)
	*diff*	*4.9*	*3.2*			
	Urban	3.5 (3.3–3.8)	2.4 (2.2–2.6)	-1.1 (-31.9)	1	1
	Rural	3.8 (3.3–4.5)	3.0 (2.5–3.6)	-0.8 (-21.1)	1.09 (0.91–1.30)	1.26 (1.03–1.54)
	*diff*	*0.3*	*0.6*			
	High education	2.7 (2.2–3.1)	1.8 (1.5–2.1)	-0.8 (-31.3)	1	1
	Middle education	3.8 (3.4–4.2)	2.6 (2.3–2.9)	-1.2 (-32.1)	1.42 (1.16–1.75)	1.41 (1.15–1.74)
	Low education	5.1 (4.6–5.8)	3.7 (3.2–4.3)	-1.4 (-27.4)	1.94 (1.58–2.39)	2.05 (1.64–2.57)
	*diff*	*2.5*	*1.9*			
Estonia	Women	2.2 (1.7–2.8)	2.1 (1.6–2.6)	-0.1 (-6.4)	1	1
	Men	14.6 (13.1–16.2)	9.3 (8.2–10.6)	-5.3 (-36.1)	6.62 (5.11–8.70)	4.52 (3.44–6.01)
	*diff*	*12.4*	*7.3*			
	Urban	6.3 (5.5–7.2)	4.4 (3.7–5.1)	-2.0 (-30.8)	1	1
	Rural	10.3 (8.9–11.8)	7.2 (6.1–8.5)	-3.1 (-29.8)	1.62 (1.34–1.97)	1.65 (1.31–2.07)
	*diff*	*3.9*	*2.8*			
	High education	2.5 (1.8–3.3)	2.9 (2.2–3.8)	0.4 (16.9)	1	1
	Middle education	8.0 (7.0–9.2)	5.8 (4.9–6.7)	-2.3 (-28.1)	3.24 (2.32–4.61)	1.99 (1.46–2.74)
	Low education	16.0 (13.3–19.1)	10.6 (8.3–13.4)	-5.4 (-33.5)	6.45 (4.52–9.35)	3.67 (2.55–5.28)
	*diff*	*13.5*	*7.7*			
Latvia	Women	4.0 (3.5–4.6)	2.2 (1.8–2.7)	-1.8 (-44.8)	1	1
	Men	22.5 (21.1–24.1)	16.7 (15.5–18.1)	-5.8 (-25.8)	5.63 (4.82–6.61)	7.57 (6.18–9.35)
	*diff*	*18.5*	*14.5*			
	Urban	9.8 (9.0–10.6)	6.8 (6.2–7.5)	-3.0 (-30.3)	1	1
	Rural	17.8 (16.2–19.4)	12.9 (11.6–14.3)	-4.9 (-27.3)	1.81 (1.60–2.05)	1.89 (1.63–2.18)
	*diff*	*8.0*	*6.1*			
	High education	5.2 (4.1–6.4)	3.8 (3.0–4.7)	-1.4 (-27.3)	1	1
	Middle education	11.6 (10.7–12.5)	8.7 (7.9–9.5)	-2.9 (-25.4)	2.24 (1.78–2.86)	2.30 (1.80–2.97)
	Low education	22.5 (19.9–25.3)	19.0 (16.2–22.1)	-3.5 (-15.7)	4.35 (3.38–5.63)	5.05 (3.82–6.72)
	*diff*	*17.3*	*15.2*			
Lithuania	Women	4.1 (3.6–4.6)	2.7 (2.3–3.1)	-1.4 (-34.1)	1	1
	Men	22.8 (21.6–24.8)	16.9 (15.9–17.9)	-5.9 (-25.9)	5.60 (4.92–6.38)	6.28 (5.41–7.30)
	*diff*	*18.7*	*14.2*			
	Urban	8.8 (8.2–9.5)	6.6 (6.1–7.1)	-2.3 (-25.6)	1	1
	Rural	21.2 (19.7–22.7)	15.3 (14.1–16.5)	-5.9 (-27.9)	2.40 (2.17–2.65)	2.32 (2.08–2.60)
	*diff*	*12.3*	*8.7*			
	High education	5.6 (4.6–6.6)	4.4 (3.7–5.2)	-1.2 (-21.4)	1	1
	Middle education	11.5 (10.7–12.3)	8.8 (8.2–9.4)	-2.7 (-23.5)	2.06 (1.71–2.51)	2.01 (1.68–2.43)
	Low education	26.2 (23.5–29.2)	20.6 (18.2–23.1)	-5.7 (-21.7)	4.73 (3.84–5.86)	4.72 (3.83–5.83)
	*diff*	*20.7*	*16.2*			

ASMR, age-standardised mortality rate per 100 000 person years; CI, confidence interval; RR, mortality rate ratio; diff, difference in ASMRs (for education between the lowest and highest level)

Living in rural areas was associated with an increased risk for drowning in the Baltic countries but not in Finland in 2000–2007; mortality rate ratios ranged from 1.6 in Estonia to 2.4 in Lithuania. However, a slightly larger mortality decline among urban residents in Finland between 2000–2007 and 2008–2015 led to increased inequalities in drowning mortality in the country. In Lithuania, the larger absolute and relative decline among rural residents resulted in diminishing absolute and relative inequalities. In Estonia and Latvia, the absolute decline was larger among rural residents but the relative change was more favourable for urban residents leading to a reduction in absolute inequalities and a small increase in relative inequalities.

Mortality rate ratios comparing low and high educated individuals were over two times larger in the Baltic countries than in Finland and ranged from 4.4 in Latvia to 6.5 in Estonia in 2000–2007. Notably, ASMRs among the high educated in Latvia and Lithuania were higher than the ASMR among the low educated in Finland. From 2000–2007 to 2008–2015, ASMRs declined in all educational groups excepting the high educated in Estonia. In all countries, the absolute decline was larger among the low educated resulting in smaller absolute inequalities. The relative decline was larger among the high educated in Finland and Latvia, among the low educated in Estonia and was about the same in all educational groups in Lithuania. As a result, relative inequalities increased in Finland and Latvia, decreased in Estonia and remained the same in Lithuania in 2008–2015.

Mutual adjustment for age, sex, urban-rural residence and educational level resulted in strongly attenuated mortality rate ratios in both periods (Table 3). Although large and statistically significant inequalities in drowning mortality persisted between men and women, and between educational groups in all countries, the mutual adjustment led to decreased educational inequalities in 2008–2015 in Finland. Urban-rural differences in drowning mortality remained statistically significant in the Baltic countries but not in Finland in 2008–2015.

**Table 3:** Adjusted mortality rate ratios for drownings in 2000–2015 in the 30–74 age group

		RR (95% CI)	
		2000–2007	2008–2015
Finland	Women	1	1
	Men	5.22 (4.36–6.25)	4.25 (3.51–5.14)
	Urban	1	1
	Rural	0.96 (0.81–1.14)	1.16 (0.96–1.40)
	High education	1	1
	Middle education	1.53 (1.26–1.86)	1.31 (1.07–1.60)
	Low education	1.75 (1.44–2.12)	1.57 (1.27–1.93)
Estonia	Women	1	1
	Men	5.79 (4.48–7.48)	3.88 (2.97–5.06)
	Urban	1	1
	Rural	1.21 (0.99–1.47)	1.34 (1.07–1.69)
	High education	1	1
	Middle education	2.85 (2.05–3.96)	1.71 (1.27–2.31)
	Low education	4.42 (3.13–6.25)	2.37 (1.69–3.34)
Latvia	Women	1	1
	Men	5.20 (4.46–6.06)	6.46 (5.31–7.87)
	Urban	1	1
	Rural	1.47 (1.30–1.66)	1.54 (1.33–1.78)
	High education	1	1
	Middle education	1.96 (1.56–2.47)	1.85 (1.45–2.36)
	Low education	2.80 (2.19–3.57)	2.90 (2.22–3.80)
Lithuania	Women	1	1
	Men	5.07 (4.46–5.75)	5.50 (4.75–6.36)
	Urban	1	1
	Rural	1.94 (1.75–2.14)	1.86 (1.67–2.08)
	High education	1	1
	Middle education	1.80 (1.49–2.18)	1.66 (1.38–1.99)
	Low education	2.84 (2.32–3.48)	2.74 (2.24–3.36)

RR, mortality rate ratio adjusted for age, sex, place of residence and educational level

## Discussion

Overall drowning ASMRs were about two to three times higher in the Baltic countries than in Finland but declined by nearly 30% in all countries between 2000–2007 and 2008–2015. Large inequalities by sex, urban-rural residence and educational level were observed in all countries in both periods. Men, rural residents and low educated individuals had substantially higher ASMRs for drowning compared to their counterparts, excepting Finland where the RR between urban and rural residents was statistically insignificant in 2000–2007. Both absolute and relative inequalities were substantially larger in the Baltic countries than in Finland. Absolute inequalities in drowning mortality declined between 2000–2007 and 2008–2015 in all countries except between urban and rural residents in Finland. Relative inequalities declined (or remained the same) between men and women in Estonia and Finland, between the low and high educated in Estonia and Lithuania, and between urban and rural residents in Lithuania, and increased in all other instances.

In EU comparisons all of the countries studied here rank well above average in terms of drowning mortality [15]. Our study further highlights that drowning mortality rates are comparatively very high in our study countries and confirms that drowning mortality is higher in the Baltic countries than Finland. Similarly, our finding that drowning mortality declined by approximately one-third in our study countries during 2000–2015 also accords with other research which has shown that drowning mortality rates have reduced globally in recent years – including in the Baltic countries and Finland [3, 14]. It is uncertain what underlies these changes but it has been suggested more generally that factors such as increased investment in water safety and changing social norms might be playing a role [3]. It is also possible that specfic measures within our study countries have helped reduce drowning ASMRs. For example, in Finland there are a variety of drowning prevention strategies in operation including community education awareness programmes, installing safety barriers and teaching public rescue skills [30]. Although there is some evidence that initatives have also taken place more recently in the Baltic countries – such as informing the general public of safe swimming places [31] – these prevention efforts have been insufficient [32] as drowning mortality in the Baltic countries continues to be high [15].

Men, those living in rural areas and people with a low education were more likely to die from drowning. Our study suggests that eductional level may play an important role in explaining the urban-rural difference in mortality from drowning. It is also possible that the same factors might be important for the observed differences across these examined exposures, in particular, alcohol. There is a large body of research evidence that alcohol consumption is an important factor in drowning deaths [33, 34] and this is also the case in our study countries. Research from Estonia showed for example, that 42% of drowning deaths involved some level of intoxication in 2015 [35], while an earlier study from Finland revealed that in the 2000–2009 period 50.6–62.0% of persons who drowned were under the influence of alcohol [36]. An autopsy-based study conducted in Lithuania in 2003–2005 showed the exceptionally large role of alcohol − 70% of females and 77% of males who drowned had consumed alcohol with the blood alcohol concentration (BAC) exceeding 2.5‰ in 58% of drowning cases [37]. Importantly, post-mortem toxicological analyses of drowning cases performed at the University of Helsinki in 2000–2009 showed that the male-to-female rate ratio in alcohol positive (BAC ≥ 50 mg/dL) cases was 7.3:1 [38], which in part, may help explain the sex gap in drowning mortality observed across our study countries where the proportion of high risk drinkers is much greater in men than women [39]. Alcohol might also play a role in the rural-urban disparity in drowning mortality. Although some research has indicated that there are few differences in the levels of urban-rural alcohol consumption in these countries [40] or that alcohol consumption is lower in rural women [41], other research has reported that rural residents not only drink alcohol more frequently than their urban counterparts, but they also drink it in larger amounts [42]. Heavier drinking may be a particularly significant driver of drowning mortality. Finally, an earlier study in Estonia indicated that an educational gradient may have existed in risky drinking during our study period as heavy episodic drinking was significantly higher in men and women with a lower education [43]. Recent research which found an inverse educational gradient in alcohol-related mortality in these countries during our study period [24] also supports the notion that alcohol might be important for the educational differences we observed in drowning ASMRs.

The finding that drowning mortality was elevated in rural areas is in line with previous research from Europe which has linked rurality to an increased risk of drowning [12] and a study from Lithuania using data from 1988 to 2009, which found that drowning mortality rates were 1.6–2.5 times higher in rural than in urban areas [44]. An earlier study showed that the vast majority of drowning deaths in our study countries occur in natural bodies of water [45] and in this context, it is possible that increased access to water [46] in conjunction with other factors such as choosing to swim in places where no other people are present [47] and reduced access to swimming lessons in rural areas [10] might also play a role in higher rural drowning mortality. Indeed, in a 2017 survey in Latvia 64% of respondents stated that they did not know how to swim or did not feel safe in the water [47].

Our finding that there was an educational gradient in drowning mortality is in line with a study from Canada which found that the hazard ratio for drowning was 1.64 in those with no high school education compared to those with a university education in a fully adjusted analysis [21]. As yet, there has been little focus on the role of socioeconomic status in drowning mortality in our study countries. However, research from other settings has indicated that education might be linked to drowning in various ways besides differences in risky alcohol use. For example, a study from Poland found that (higher) parental education was associated with better swimming competency in young adults [48], while other research has also shown that having a private education in childhood also results in better swimming and water safety skills [49]. Higher education has also been linked to lower odds for engaging in health-risk behaviours in older adults [50] and it is possible that this might extend to the aquatic environment, although a recent systematic literature review found that education was not associated with lifejacket use in children and adults [51].

Absolute inequalities in drowning mortality fell in the Baltic countries across the study period while the changes in relative inequalities were more variable. Without more detailed research within the countries it is difficult to speculate what underlies these results. As there is some indication that overall alcohol consumption rose across the study period in the Baltic countries but remained more constant in Finland [52] it can be speculated that alcohol may have played less of a role in these observed changes. It is possible that these changes are also part of longer-term trends. For example, research from Finland has shown that drowning deaths have been falling since the 1950s [36], during which time education levels have increased (see Table 1), while the prevalence of being able to swim is seemingly much higher in younger compared to older age groups [53]. More research is thus needed to determine how longer-term social trends might have impacted absolute and relative inequalities in drowning mortality in our study countries.

### Strengths and limitations

The main strength of this study is the use of highly comparable register-based data with reliable individual-level information on attained educational level and urbanicity. In addition, since the early 2000s the Baltic countries have participated in the Nordic collaboration to coordinate coding and classification practices of causes of death and thus improve the comparability of mortality statistics in the region [54]. However, the study also has several limitations that should be considered. First, this study used data on unintentional drowning and submersion but did not include information on drowning deaths due to other causes such as floods and water transport accidents. In addition, we also lacked information on the type of body of water in which the deceased drowned. Such information would have helped us to better understand the phenomenon of drowning in these countries. Second, the finding that the urban-rural difference in drowning mortality was considerably lower in Finland compared to the differences observed in the Baltic countries might in part, be explained by the fact that urban and rural areas were defined in a different way in Finland, thus complicating comparisons between the countries. Third, to ensure the accuracy of the educational attainment data we limited the age range in this study to 30–74 years old. However, it should be noted that there is evidence that a large number of drowning deaths also occur among children, younger adults and those in the 75 + age group [18] which should also be a focus for future research. Finally, the exclusion of register-only-based data in the Baltic countries could be a potential source of bias yet results from a supplementary sensitivity analysis we performed showed that excluding register-only-based data had only a marginal effect on overall drowning mortality in Latvia (see Supplementary Table 1). Although we cannot exclude the possibility that the effect differed by educational level or urban-rural residence, we believe that this bias is unlikely to have had any major effect on our conclusions related to drowning mortality and its changes in Latvia.

## Conclusions

This study showed that although there was a sharp fall in drowning mortality in all studied countries in 2000–2015 there were also noticeable differences with higher overall drowning mortality and larger inequalities in drowning mortality observed in the Baltic countries than in Finland. Moreover, despite the sharp drop in mortality, drowning ASMRs still remained very high in comparative terms at the end of the study period – especially in the Baltic countries. This suggests that a concerted effort needs to be made to reduce drowning mortality in these countries and that further research needs to be undertaken so that interventions can be targeted at those most at risk of drowning. For example, measures such as teaching school-age children swimming and water safety skills [1] may be essential for reducing drowning deaths further in the future, especially as many children in these countries are still not able to swim, while those that can, sometimes overestimate their own competency in the water [55]. In addition, given the large role that alcohol plays in adult drowning deaths in these countries [56] and also possibly, in inequalities in drowning mortality, a focus on alcohol in drowning prevention efforts may also be important in this setting. Finally, this study used data from 2000 to 2015. Further follow-up studies using more recent and detailed data are required to understand the current situation and address the deficits of this study.

## Electronic supplementary material

Below is the link to the electronic supplementary material.

Supplementary Material 1

## Data Availability

Due to the data protection regulations of the respective national statistical offices we are not allowed to make the data available to third parties. Interested researchers have the possibility to obtain data by contacting national statistical offices directly (Statistics Estonia email: stat@stat.ee; Statistics Lithuania email: statistika@stat.gov.lt; the Central Statistical Bureau of Latvia email: pasts@csb.gov.lv; Statistics Finland email: tutkijapalvelut@stat.f). Alternatively, contact the authors from the respective countries to enquire about potential research collaboration.
