# Supplementary material for: Sociodemographic inequalities in mortality from drowning in the Baltic countries and Finland in 2000–2015: a register-based study
Source: BMC Public Health. 2023 Jun 7;23:1103. doi: 10.1186/s12889-023-15999-9 (PMC10245523; doi:10.1186/s12889-023-15999-9)
Supplement: Supplementary file 1 — Supplementary Material 1 [file 12889_2023_15999_MOESM1_ESM.docx]

**Supplementary Table 1** Impact of excluding register-only-based census records on drowning mortality among 30–74 year olds in Latvia, 2000–2015

| Period | Census + registry | Census |  | Census + registry | Census |
| --- | --- | --- | --- | --- | --- |
|  | ASMR (95% CI) | ASMR (95% CI) | *P* value | RR (95% CI) | RR (95% CI) |
|  |  |  |  |  |  |
| 2000–2007 | 12.7 (12.0–13.4) | 12.5 (11.8–13.3) | 0.660 | 1 | 1 |
| 2008–2015 | 9.3 (8.7–9.9) | 9.2 (8.5–9.8) | 0.834 | 0.73 (0.67–0.80) | 0.73 (0.67–0.80) |
|  |  |  |  |  |  |

ASMR, age-standardised mortality rate per 100 000 person years; CI, confidence interval.

RR, rate ratios comparing the 2008–2015 period with the 2000–2007 period (reference category).

*P* values are for the differences resulting from excluding register-only-based census records.
